# CAPN 7 promotes the migration and invasion of human endometrial stromal cell by regulating matrix metalloproteinase 2 activity

**DOI:** 10.1186/1477-7827-11-64

**Published:** 2013-07-15

**Authors:** Hongyu Liu, Yue Jiang, Xiaoyan Jin, Lihua Zhu, Xiaoyue Shen, Qun Zhang, Bin Wang, Junxia Wang, Yali Hu, Guijun Yan, Haixiang Sun

**Affiliations:** 1 Reproductive Medicine Center, The Affiliated Drum Tower Hospital of Nanjing University Medical School, Nanjing 210008, Jiangsu, China

**Keywords:** CAPN 7, Endometrial stromal cell, Migration, Invasion

## Abstract

**Background:**

Matrix metalloproteinase 2 (MMP-2) has been reported to be an important regulator of cell migration and invasion through degradation of the extracellular matrix (ECM) in many diseases, such as cancer and endometriosis. Here, we found calcium-activated neutral protease 7 (CAPN 7) expression was markedly upregulated in the eutopic endometrium and endometrial stromal cells of women diagnosed with endometriosis. Our studies were carried out to detect the effects of CAPN 7 on human endometrial stromal cell (hESC) migration and invasion.

**Methods:**

Western blotting and quantitative real-time PCR were used to detect the expression of CAPN 7 in endometriosis patients and normal fertile women. Scratch-wound-healing and invasion chamber assay were used to investigate the role of CAPN 7 in hESC migration and invasion. Western blotting, quantitative real-time PCR and zymography were carried out to detect the effect of CAPN 7 on the expressions and activity of MMP-2.

**Results:**

CAPN 7 was markedly up-regulated in endometriosis, thereby promoting the migration and invasion of hESC. CAPN 7 overexpression led to increased expression of MMP-2 and tissue inhibitor of metalloproteinases 2 (TIMP-2); CAPN 7 knockdown reversed these changes. CAPN 7 increased MMP-2 activity by increasing the ratio of MMP-2 to TIMP-2. We also found that OA-Hy (an MMP-2 inhibitor) decreased the effects of CAPN 7 overexpression on hESC migration and invasion by approximately 50% and 55%, respectively. Additionally, a coimmunoprecipitation assay demonstrated that CAPN 7 interacted with activator protein 2α (AP-2α): an important transcription factor of MMP-2.

**Conclusions:**

CAPN 7 promotes hESC migration and invasion by increasing the activity of MMP-2 via an increased ratio of MMP-2 to TIMP-2.

## Background

Endometriosis is a common gynecological disease characterized by the presence of endometrial tissue outside the uterine cavity [1]. This disease affects approximately 10% of women of reproductive age and is associated with pelvic pain, dysmenorrhea and infertility [1,2]; however, the exact pathogenesis remains unclear. It has been shown that the eutopic endometrial stromal cell migration rate is higher in cells from endometriosis patients compared to endometriosis-free controls [3]. Several studies have shown that certain genes and proteins in the endometrium, including p-ERK [3], DJ-1 [4] and MMP-2 [5], are involved in endometriosis-associated proliferation, migration, invasion and angiogenesis. The aberrant expression of these proteins is a key factor in endometriosis pathogenesis.

Calpains are a family of calcium-dependent cysteine proteases that consists of more than ten mammalian gene products that are divided into two categories: classical calpains and non-classical calpains [6]. Calpains have been suggested to play important roles in many biological processes, including apoptosis and migration [7,8]. Aberrant calpain expression or activity is often related to serious disorders. CAPN 7, a non-classical calpain, lacks the EF-hand domain, and thus, its activity does not depend on Ca^2+^[9]; however, the exact structure and pathological significance of CAPN 7 have not been fully elucidated.

In our study, we found that CAPN 7 mRNA and protein expression was upregulated in the eutopic endometrium and endometrial stromal cells from women who were diagnosed with endometriosis, and we further investigated the effects of CAPN 7 on hESC motility and invasion.

## Methods

### Isolation and culture of human endometrial stromal cells

HESCs were isolated from endometrial tissue obtained via endometrial biopsy from normal fertile women with regular menstrual cycles (n = 25) and eutopic endometrial stromal cells were isolated from eutopic endometrial of patients with pelvic endometriosis (n = 7). All the endometriosis patients were diagnosed by the laparoscopy and the age of the patients were 25 to 35 years old. The Drum Tower Hospital Research and Ethics Committee approved this study, and all of the patients gave their informed consent. The tissues were immediately placed into culture medium and processed according to Sun et al. [10], with minor modifications. First, the endometrial tissues were minced and enzymatically digested with 0.1% (w/v) collagenase I (Worthington, Freehold, NJ, USA) for 30 min at 37°C. Next, the digested tissues were filtered through 30 μm-sieve gauze to separate the stromal cells from the glands. The endometrial stromal cells were maintained in DMEM/F12 supplemented with 10% (v/v) FBS, 50 IU/mL of penicillin and 50 μg/mL of streptomycin (Gibco BRL/Invitrogen, Carlsbad, CA, USA), seeded into culture dishes and incubated at 37°C in 5% CO_2_. The cultured stromal cells were 95% pure, as determined by vimentin staining.

### Adenovirus construction

An adenovirus construct bearing the human full-length CAPN 7 gene (Ad-Flag-CAPN 7) was produced using AdMax (Microbix, Mississauga, Ontario, Canada). An adenovirus bearing LacZ (Ad-LacZ) was obtained from BD Biosciences Clontech (Palo Alto, CA, USA). The viruses were packaged and amplified in HEK293A cells and purified using CsCl banding, followed by dialysis against 10 mmol/L Tris-buffered saline with 10% (w/v) glycerol. Titration was performed in HEK293A cells with the Adeno-X Rapid Titer kit (BD Biosciences Clontech) according to the manufacturer’s instructions. HESCs were infected with Ad-LacZ or Ad-Flag-CAPN 7 at an MOI of 50.

### siRNA knockdown assays

A pair of small interfering (si)RNA oligonucleotides specific for human CAPN 7 (sense strand: 5′-CAUUAGUGGUUUCUCAAUAdTdT-3′ and 3′-dTdT GUAAUCACCAAAGAGUUAU-5′) and a pair of control siRNA oligonucleotides were synthesized by RIBOBIO (Guangzhou, China). HESCs were grown to 70-80% confluence and transfected with siRNAs with the SuperFectTM^II^ (Pufei Biotech, Shanghai, China) transfection reagent at a final concentration of 50 nM according to the manufacturer’s recommendations.

### Cell migration and invasion assays

The wound and invasion assays were performed according to Rai et al. [4], with minor modifications. For the scratch wound assay, cells were starved in DMEM/F12 plus 2.5% (v/v) FBS for 24 hours before the assay and the cells were maintained in DMEM/F12 plus 2.5% (v/v) FBS for the entire experiment. When the cells reached 80-90% confluence, the hESCs were infected with adenovirus at 50 MOI or transfected with 50 nM siCTL or 50 nM siCAPN 7. The confluent cell monolayer was wounded with a 200 μL plastic cell scraper at the 0 h time point (immediately after siRNA transfection or 6 h after adenovirus infection). Cell migration was evaluated at the wound front at 0, 24, 48 and 72 h after wounding. For the matrigel-coated invasion chamber assay, cells were also starved in DMEM/F12 plus 2.5% (v/v) FBS for 24 hours and then infected with Ad-LacZ or Ad-Flag-CAPN 7 at an MOI of 50 or transfected with siCTL or siCAPN 7 at a final concentration of 50 nM. Polycarbonate membrane filters (8 μm pore size) (Millipore Corporation, Billerica, MA, USA) were pre-coated with matrigel. Next, the cells (5 × 10^4^ cells per chamber) in 100 μL DMEM/F12 plus 0.1% (w/v) BSA were added to the top chambers. The bottom chambers were filled with 700 μL DMEM/F12 supplemented with 10% (v/v) FBS and then incubated at 37°C for 72 h. Then, the invasion cells were fixed, dyed and counted. For inhibitor experiments, OA-Hy (Millipore Corporation, Billerica, MA, USA) was added at a concentration of 20 μM 1 h before adenovirus infection; the OA-Hy concentration was maintained throughout the experiment. All the experiments were performed at three times and each experiment was performed with cells isolated from three patients (hESC were isolated from normal fertile women and the cultured stromal cells were 95% pure, n = 9).

### Western blotting

Total proteins were extracted and analyzed via western blotting as described previously [11]. HESCs and endometrium were lysed in lysis buffer (50.0 mmol/L Tris, pH 7.6, 150.0 mmol/L NaCl, 0.1% (w/v) SDS, 1.0% (w/v) NP-40, protease inhibitor cocktail and phosphatase inhibitor cocktail (Sigma, St. Louis, MO, USA)). The protein concentrations in the total lysates were determined using the Bradford assay (Bio-Rad Laboratories, Hercules, CA, USA). Equal amounts of protein (30 μg) were separated on 10% (v/v) sodium dodecyl sulfate (SDS)–polyacrylamide gel by electrophoresis for 1.5 h, then transferred to a polyvinylidene fluoride membrane (Millipore, Billerica, MA, USA) and probed with the following antibodies as appropriate: anti-CAPN 7 (1:1000 dilution; Santa Cruz Biotechnology, CA, USA, SC-50501), anti-MMP-2 (1:1000 dilution; Bioworld Technology, MN, USA, BS1236), anti-Flag-HRP (1:2000 dilution; Sigma, St. Louis, MO, USA, A8592) and anti-β-actin (1:5000 dilution; Abcam, Cambridge, MA, USA, AP0060). An enhanced chemiluminescence kit (Amersham Biosciences Corp., Piscataway, NJ, USA) was used to visualize the blots.

### Co-immunoprecipitation

Precipitations were performed as previously described [12]. Briefly, 600 μg of proteins was immunoprecipitated with anti-rabbit IgG or anti-AP2α (Bioworld Technology, MN, USA, BS1015) at 4°C for 12 h. The washed precipitates were detected by western blotting as described above.

### Quantitative real-time PCR

Total RNAs were isolated using the TRIzol reagent (Invitrogen, Carlsbad, CA, USA) according to the manufacturer’s instructions. Two micrograms of total RNA were subsequently reverse transcribed in a total volume of 25 μL at 37°C for 1 hour to produce cDNA, and SYBR Green fluorescence was measured as described previously [11]. The reactions were performed using a MyiQ Single Color Real-time PCR detection system (Bio-Rad) for 40 cycles (95°C for 15 s, 55°C for 30 s, and 72°C for 30 s) after an initial 3 min incubation at 95°C. The specific primers used for 18S rRNA detection were 5′-CGGCTACCACATCCAAGGAA-3′ and 5′-CTGGAATTACCGCGGCT-3′, the CAPN 7 primers were 5′-ATGGTGTCCCAAGAAAGGTG-3′ and 5′-TGGTATCCAGCCAGTCAGTG-3′, the MMP-2 primers were 5′-ACATCAAGGGCATTCAGGAG-3′ and 5′-ATCTCACCACGGATCTGAAC-3′, and the TIMP-2 primers were 5′-CCAAGCAGGAGTTTCTCGAC-3′ and 5′-GACCCATGGGATGAGTGTTT-3′. The efficacy of CAPN 7, MMP-2 and TIMP-2 is 90.4%, 92.7% and 100.8% respectively and the expression level of each gene was normalized against the internal reference gene 18S to detect fold changes in expression. Next, melting curve and agarose gel electrophoresis analyses were used to confirm the specificity of the obtained PCR products and the real-time PCR results.

### MMP-2 zymography

Gelatin zymography was performed as previously described with some modifications [13]. Supernatants were collected from hESCs treated with adenovirus or siRNA for 48 h in DMEM/F12 plus 2.5% (v/v) c-FBS. Equal amounts of protein (10 μg) were separated on 10% (v/v) SDS-polyacrylamide gels that contained gelatin by electrophoresis for 4 h, then the gels were washed with 2.5% (v/v) Triton X-100 (2 × 20 min). Next, the gels were incubated in a post-electrophoretic buffer (50 mmol/L Tris, 5 mmol/L CaCl_2_, 200 mmol/L NaCl, and 3% (w/v) Brij-35) at 37°C for 36 h and stained with 0.125% (w/v) coomassie brilliant blue for 1 h. Finally, the gels were destained in 30% (v/v) methanol/10% (v/v) glacial acetic acid. Various MMPs were distinguished according to their molecular weights.

### Statistical analysis

All experiments in this study were performed at least three times. Statistical analysis was performed with ANOVA, followed by the Student–Newman-Keulsmultiple comparisons test. P < 0.05 was considered statistically significant.

## Results

### CAPN 7 expression is increased in endometriosis

**Figure 1:**
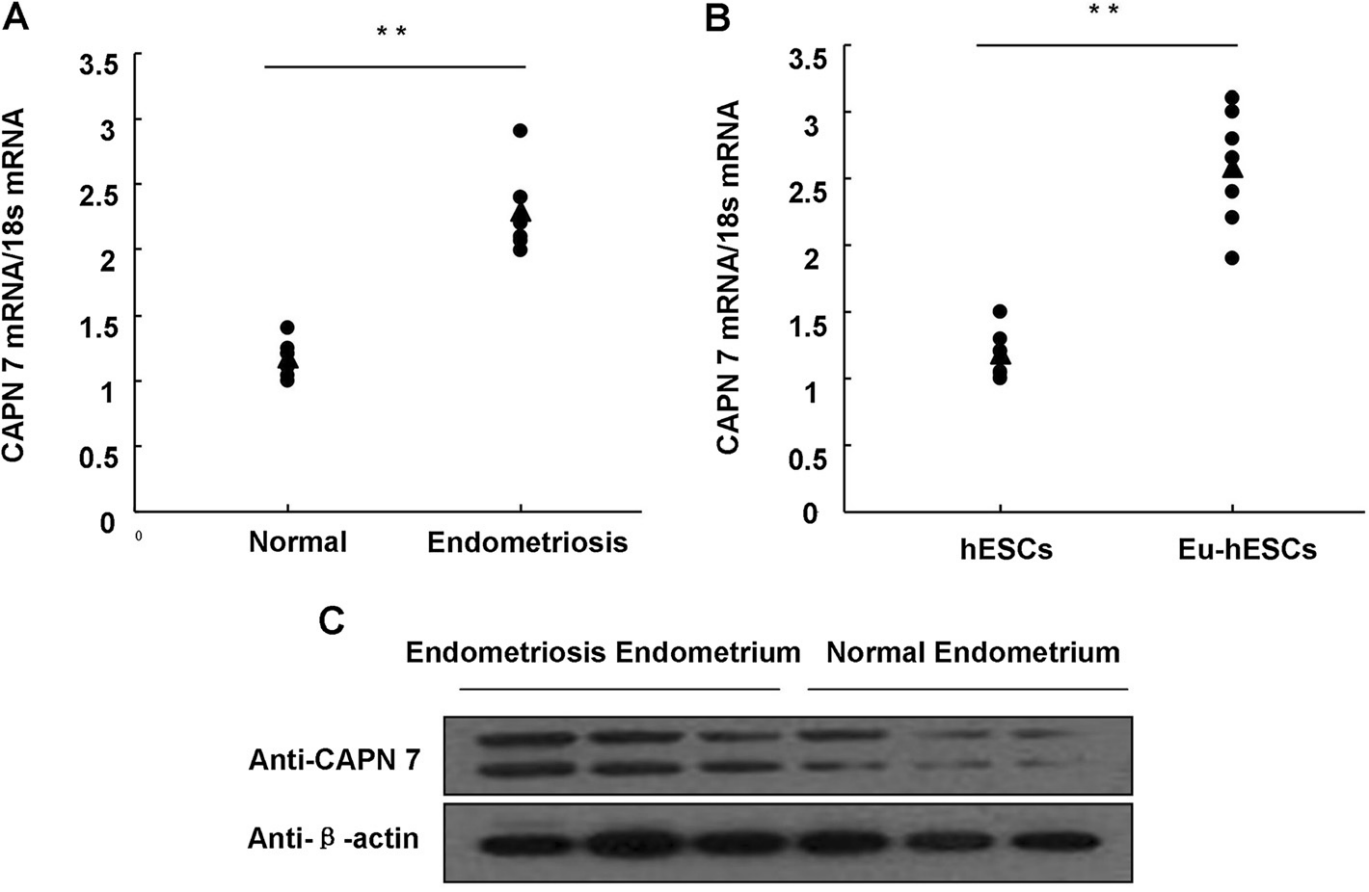
**CAPN 7 expression in endometriosis. (A)** CAPN 7 expression in the eutopic endometrium from women with endometriosis and endometrial from normal fertile controls was measured using quantitative real-time PCR; * * P < 0.01 compared to the endometrium from normal fertile women (n = 7, triangles indicate the median expression in each group). **(B)** CAPN 7 expression in the eutopic endometrium stromal cells from women with endometriosis (Eu-hESCs) and hESCs from normal fertile controls was measured using quantitative real-time PCR; * * P < 0.01 compared to normal fertile women. (n = 7, triangles indicate the median expression in each group). **(C)** Western blotting results showed higher CAPN 7 protein expression in the eutopic endometrium of women with endometriosis (n = 3).

To determine the role of CAPN 7 in endometriosis, we first examined CAPN 7 expression in endometriosis patients using western blotting and quantitative real-time PCR. CAPN 7 expression was significantly higher in the eutopic endometrium and endometrial stromal cells from endometriosis patients than normal fertile subjects at both the mRNA and protein level; CAPN 7 mRNA levels were increased by more than 2-fold in endometriosis patients (Figure 1).

### CAPN 7 affects hESC migration and invasion

**Figure 2:**
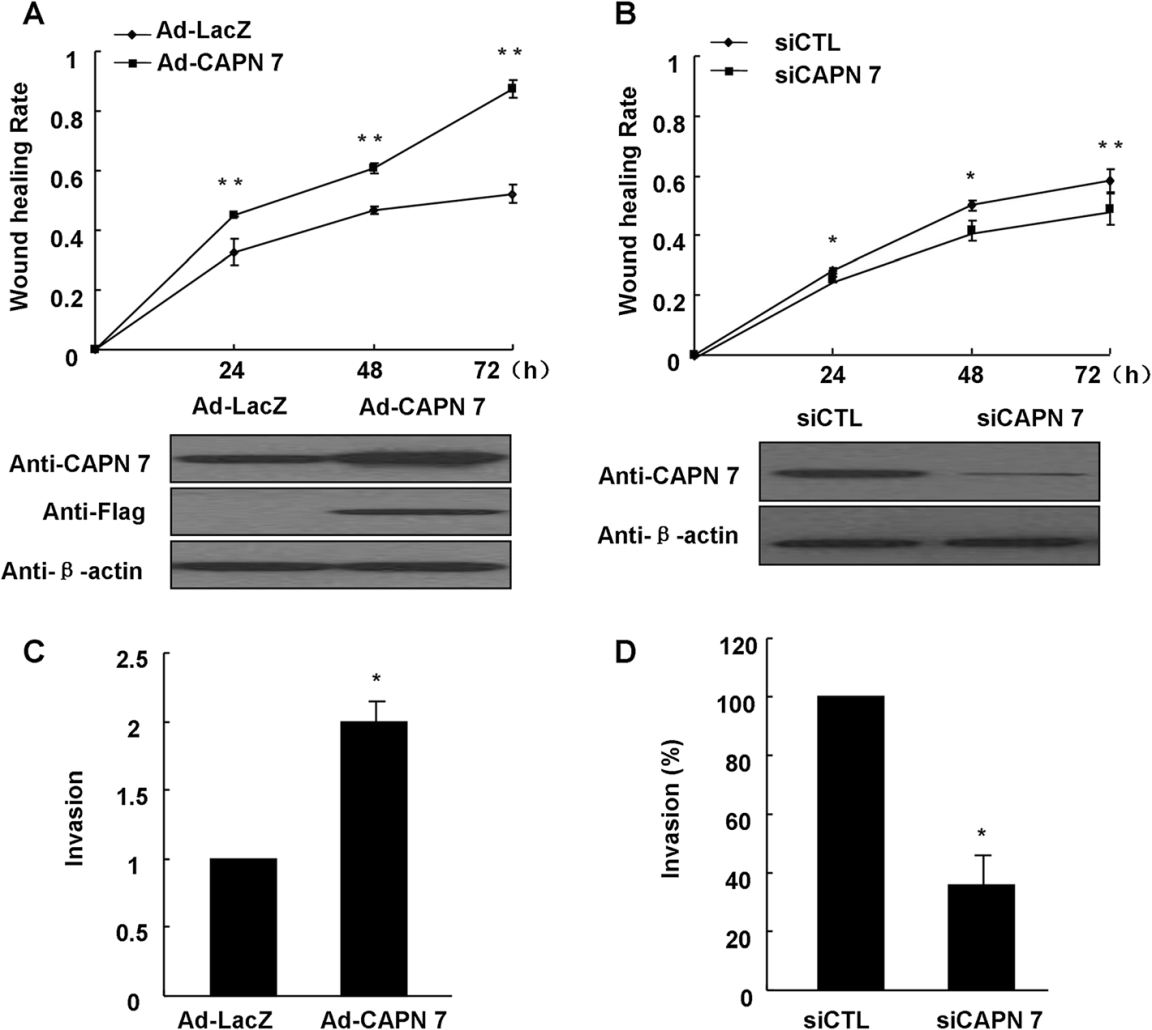
**CAPN 7 affects hESC migration and invasion. (A)** HESC were infected with Ad-LacZ or Ad-Flag-CAPN 7 (MOI = 50). The migration rates (migration distance from the 0 h time point to a certain time) of the two groups are shown in the line graph; **P < 0.01 compared with Ad-LacZ. **(B)** Migration rates of hESC transfected with control siRNA (siCTL) or siRNA specific for CAPN 7 (50 nM); * *P* < 0.05, **P < 0.01 compared with siCTL. **(C)** CAPN 7 overexpression increased cell invasion by approximately 2-fold, * *P* < 0.05 compared to Ad-LacZ. **(D)** Knockdown of endogenous CAPN 7 expression decreased cell invasion by approximately 65% compared with siCTL (* *P* < 0.05). CAPN 7 protein expression was measured by western blotting (hESC were isolated from normal fertile women, n = 9).

A wound-healing assay was performed to detect the effect of CAPN 7 on hESC migration. Adenovirus-mediated CAPN 7 overexpression significantly increased hESC migration rates. Compared to the corresponding control, the migration rates increased by 1.2-, 1.3- and 1.7-fold after infection with the CAPN 7 adenovirus at 24, 48 and 72 h, respectively (Figure 2A). However, after CAPN 7 knockdown, the migration rates decreased by 10% compared to the control at all time points (Figure 2B). CAPN 7 protein expression in the cells was further confirmed via western blotting after the wound-healing assay. We also found that CAPN 7 overexpression increased the invasiveness of hESC by approximately 2-fold in a matrigel basement invasion assay (Figure 2C). However, CAPN 7 knockdown decreased invasiveness by approximately 65% compared with hESC exhibiting normal CAPN 7 expression (Figure 2D).

### CAPN 7 overexpression upregulates MMP-2 expression and activity in hESC

**Figure 3:**
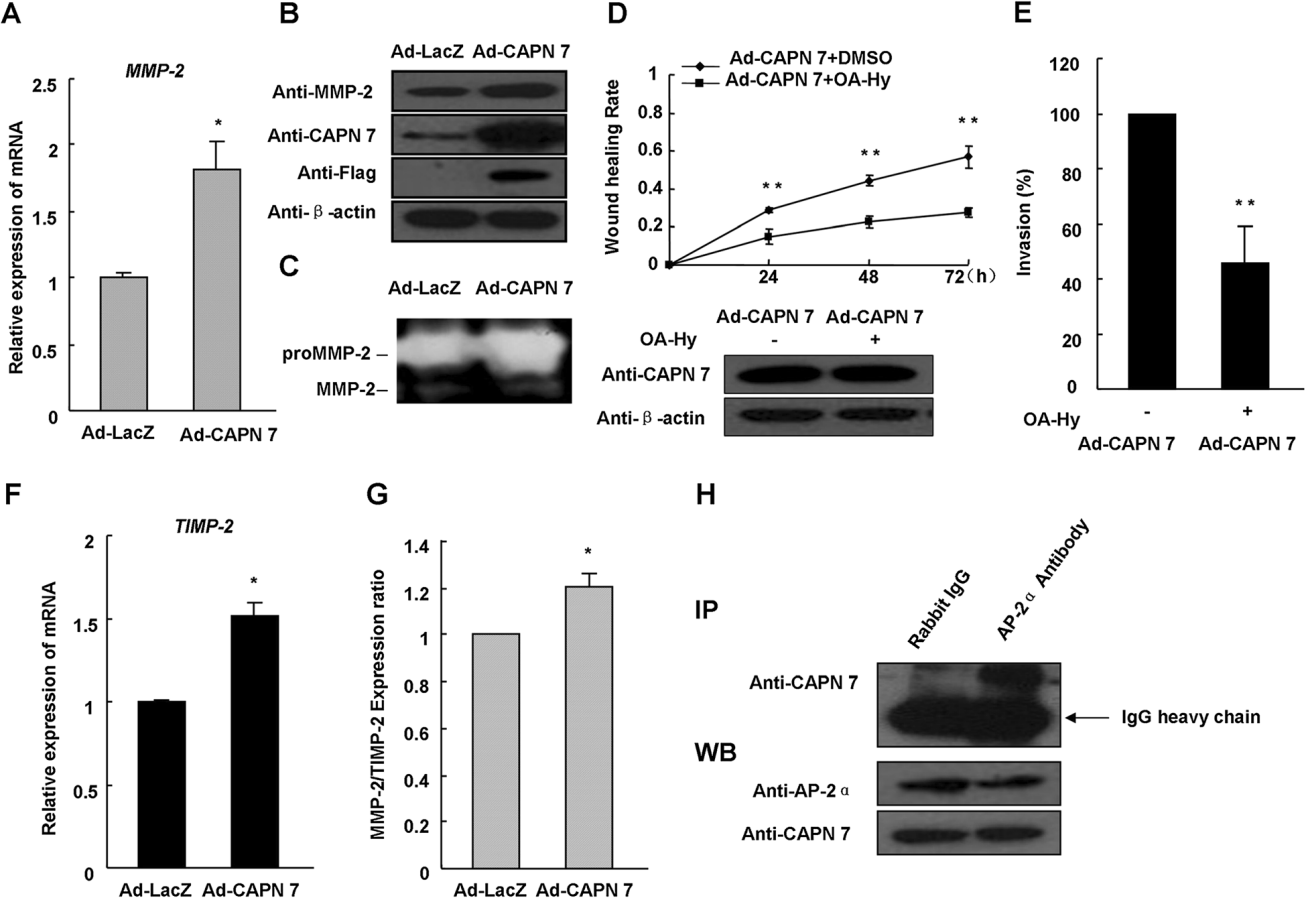
**CAPN 7 overexpression upregulates MMP-2 expression and activity in hESC. (A)** MMP-2 mRNA expression was measured using real-time PCR following CAPN 7 overexpression; * *P* < 0.05 compared to Ad-LacZ. **(B**, **C)** MMP-2 protein expression and activity were measured using western blotting and zymography, respectively, following CAPN 7 overexpression. **(D)** OA-Hy decreased the migratory effects of CAPN 7 overexpression on hESC by approximately 50% at all time points; ** *P* < 0.01 compared to controls. **(E)** OA-Hy decreased the invasion ability of CAPN 7 overexpression in hESC by approximately 55%; ** *P* < 0.01 compared to the control. **(F)** TIMP-2 mRNA expression was measured using real-time PCR following CAPN 7 overexpression; * *P* < 0.05 compared to Ad-LacZ. **(G)** The ratio of MMP-2 to TIMP-2 mRNA expression was measured under the same conditions, * *P* < 0.05 compared to controls. **(H)** A coimmunoprecipitation assay was used to detect interactions between CAPN 7 and AP-2α (IP: immunoprecipitation; WB: western blotting) (hESC were isolated from normal fertile women, n = 18).

ECM degradation by matrix metalloproteinases (MMPs) is required for cell migration and invasion. MMP-2 is one of the major proteinases that play this role in the human endometrium [5]. Our results suggested that CAPN 7 overexpression increased both the expression (mRNA increased by 1.8-fold; Figure 3A and 3B) and the activity of MMP-2 (Figure 3C). Furthermore, we found that OA-Hy (an MMP-2 inhibitor) decreased the effects of CAPN 7 overexpression on hESC migration and invasion by approximately 50% and 55%, respectively (Figure 3D and 3E). To further quantify the role of CAPN 7, we examined TIMP-2 expression (endogenous inhibitor of MMP-2) and found that CAPN 7 overexpression increased the expression of TIMP-2 mRNA by 1.5-fold (Figure 3F) and increased the MMP2/TIMP-2 ratio in hESC (Figure 3G). These results suggest that CAPN 7 affects the activity of MMP-2 by disrupting the balance between MMP-2 and TIMP-2, thereby regulating hESC migration and invasion. Additionally, a coimmunoprecipitation assay showed that CAPN 7 interacted with AP-2α (Figure 3H), suggesting that CAPN 7 regulates MMP-2 mainly at the transcriptional level. However, an in-depth study is required.

### Knockdown of CAPN 7 expression decreases MMP-2 expression and activity in hESC

**Figure 4:**
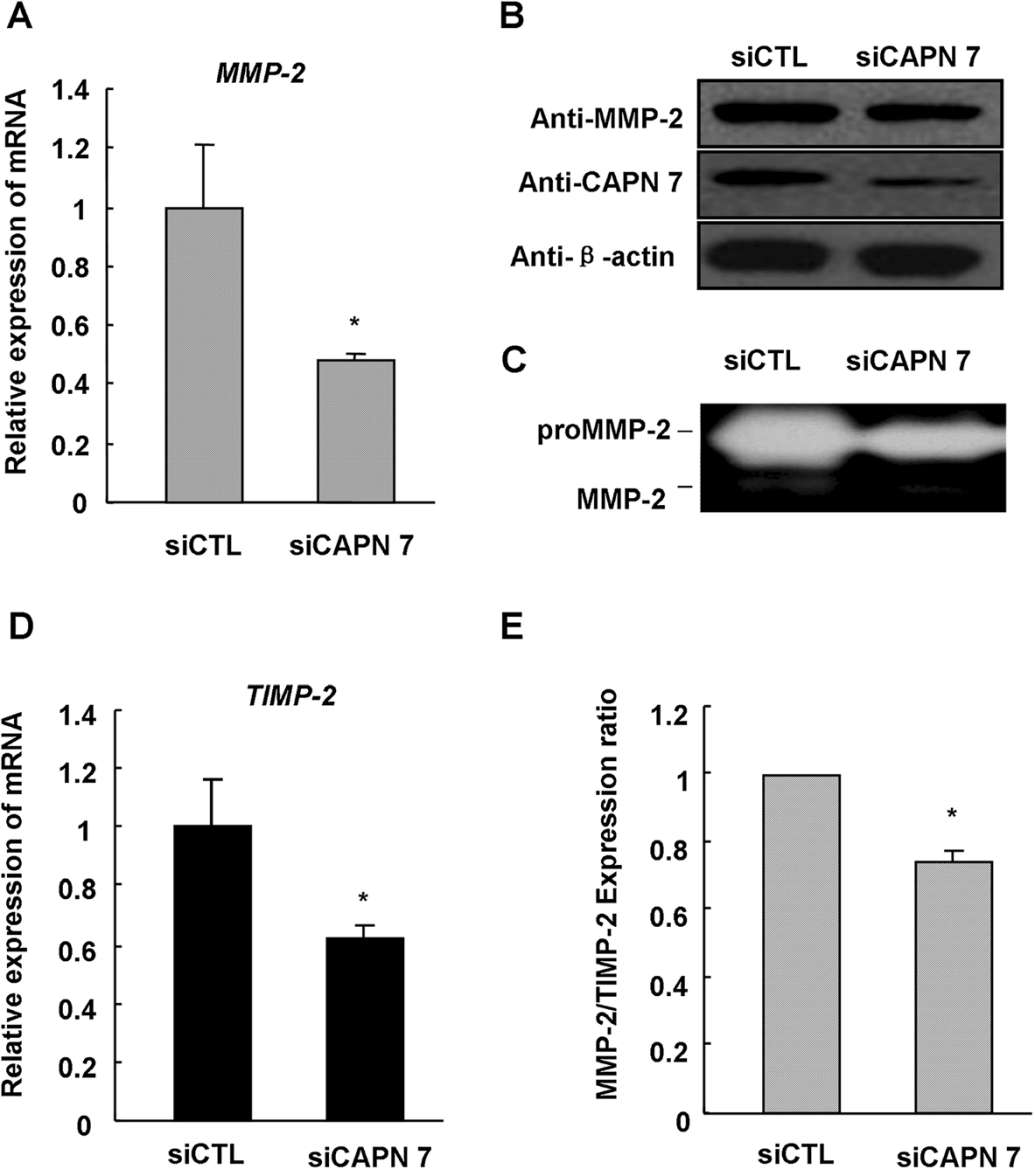
**CAPN 7 knockdown decreases MMP-2 expression and activity in hESC. (A)** MMP-2 mRNA expression was measured following CAPN 7 knockdown; * *P* < 0.05 compared to siCTL. **(B**, **C)** MMP-2 protein expression and activity were measured under the same conditions. **(D)** TIMP-2 mRNA expression was measured following CAPN 7 knockdown; * *P* < 0.05 compared to siCTL. **(E)** The ratio of MMP-2 to TIMP-2 mRNA expression was measured following CAPN 7 knockdown; * *P* < 0.05 compared to controls (hESC were isolated from normal fertile women, n = 9).

For an in-depth understanding of how CAPN 7 regulates hESC migration and invasion, we knocked down endogenous CAPN 7 expression in hESC. CAPN 7 knockdown reduced both the expression (mRNA decreased by 50%; Figure 4A and 4B) and the activity of MMP-2 (Figure 4C). Furthermore, quantitative real-time PCR also indicated that CAPN 7 knockdown reduced the expression of TIMP-2 mRNA by 40% (Figure 4D). We observed that the inhibitory effect of CAPN 7 knockdown on MMP-2 expression was greater than the effect on TIMP-2 (Figure 4E).

## Discussion

In this report, we identified the CAPN 7 regulation of MMP-2 to promote hESC migration and invasion. First, we showed that CAPN 7 expression is increased in endometriosis. CAPN 7 is a member of the calpains family, which is composed of enzymes whose activities depend on calcium [6]. Calpains have been implicated in many cellular processes, such as apoptosis and migration [7,8]. Aberrant calpain expression is related to a variety of diseases. For example, loss of function of the full-length isoform of calpain 3 results in limb girdle muscular dystrophy type 2A [13], knocking out alleles of mouse CAPN 8 and CAPN 9 results in ethanol-induced gastric injury [14], and aberrant calpain 10 activation results in type 2 diabetes [15]. Most importantly, the expression of calpain 6, a non-classical calpain, is increased in leiomyosarcomas [16], while the expression of calpain 5 is decreased in endometriosis [17].

Recent studies have underlined the critical role of MMP-2 in endometriosis [5]. MMP-2 belongs to the MMP family protein; these proteins play important roles in the processes of migration and invasion [18]. Our results suggested that CAPN 7 affects MMP-2 activity by modifying the ratio of MMP-2/TIMP-2. It was previously reported that eutopic uterine endometrium from endometriosis patients had higher MMP-2 and lower TIMP-2 expression levels compared with normal fertile women [5]. Our results are not completely consistent with these reports. However, an imbalance between MMP and TIMP expression was found to be important for MMP activity and was involved in various medical conditions, including liver fibrosis and endometriosis [5,19,20]. Other studies have reported that TIMP-2 is unique, as it functions as both an MMP inhibitor and activator [21], meaning that it activates MMP-2 at low concentrations but inhibits MMP-2 at high concentrations [22]. It is possible that CAPN 7 promoted TIMP-2 expression to a low concentration and disrupted the balance between MMP-2 and TIMP-2, leading to upregulated MMP-2 activity. A previous report showed that calpain 1 and calpain 2 were involved in the adhesive and invasive abilities of osteosarcoma cells by affecting MMP-2 secretion [23]. Another study suggested that calpain 2 could affect glioblastoma cell invasion by regulating MMP-2 activity [24]. Here, we showed that CAPN 7 promotes hESC migration and invasion by increasing the activity of MMP-2; thus, it is plausible to speculate that CAPN 7 might play an important role in endometriosis.

Our findings suggested that CAPN 7 regulates MMP-2 mainly at the transcriptional level. AP-2α is a member of the AP-2 family and was previously reported to affect the transcription of many genes involved in invasion and adhesion [25]. AP-2α was demonstrated to enhance cell invasion by promoting MMP-2 activation [26]. Furthermore, a study on nerve growth factor reported that the AP-2-binding site was important for nerve growth factor-induced MMP-2 promoter activity [27]. Reports have indicated that calpains can degrade several transcription factors, including NF-κB [28], STAT6 [12] and P53 [29], and thus play roles in many diseases. However, whether CAPN 7 affects MMP-2 expression by degrading AP-2α will be the subject of our further studies. Additionally, we analyzed the expression of matrix metalloproteinase 9 and tissue inhibitor of metalloproteinases 1 mRNA under the same conditions, but the results suggested that CAPN 7 had little effect on the expression of these genes (data not shown).

## Conclusions

The present study provides the first evidence that CAPN 7 is highly expressed in endometriosis. Increased CAPN 7 expression promoted the migration and invasion of hESC by increasing the activity of MMP-2 via the increased ratio of MMP-2 to TIMP-2. Elucidating the mechanisms that underlie aberrant CAPN 7 expression in endometriosis could be of interest with regard to a better understanding of endometriosis pathogenesis and, ultimately, the development of new diagnostic tools and targeted therapies.

## Abbreviations

AP-2α: Activator protein 2α; CAPN 7: Calcium-activated neutral protease 7; C-FBS: Charcoal-stripped fetal bovine serum; ECM: Extracellular matrix; FBS: Fetal bovine serum; hESC: Human endometrial stromal cell; h: Hour; MMP-2: Matrix metalloproteinase 2; MMPs: Matrix metalloproteinases; MOI: Multiplicity of infection; SDS: Sodium dodecyl sulfate; siRNA: Small interfering RNA; TIMP-2: Tissue inhibitor of metalloproteinases 2.

## Competing interests

The authors declare that they have no competing interests.

## Authors’ contributions

GY, YH and HS were responsible for the conception and design of the study. HL, YJ, XJ, LZ, XS, QZ, BW and JW were responsible for acquisition of data. HL, YJ, XJ, HS and GY, performed the data analysis and drafted the manuscript. All authors participated in interpretation of the findings. YH, HS and GY revised and commented the draft, and all authors read and approved the final version of the manuscript. All authors confirm that the content has not been published elsewhere and does not overlap with or duplicate their published work. All authors read and approved the final manuscript.
